# MCAM (CD146) Gene Encodes Chicken Blood Alloantigen System H

**DOI:** 10.3390/genes17040412

**Published:** 2026-03-31

**Authors:** Janet E. Fulton, Amy M. McCarron, Anna Wolc, Brandi A. Sparling, Lowdan Ali, Courtney Jaeger, Robert L. Taylor

**Affiliations:** 1 Hy-Line International, Research and Development, P.O. Box 310, Dallas Center, IA 50063, USA; chknphd@outlook.com (J.E.F.); awolc@iastate.edu (A.W.);; 2 Department of Animal Science, Iowa State University, Ames, IA 50011, USA; 3 College of Veterinary Medicine, Western University of Health Sciences, Pomona, CA 91766, USA; bsparling@westernu.edu; 4 School of Agriculture and Food Systems, West Virginia University, Morgantown, WV 26506, USA

**Keywords:** H alloantigen, MCAM, CD146, chicken H blood system

## Abstract

Background/Objectives: Alloantigen H is one of thirteen systems in the chicken. Little is known about this system which has two serological alleles. The objectives of this study were (1) to identify the genetic region encoding the chicken alloantigen H, and (2) to develop DNA detection-based methods to aid H system allele identification. Methods: SNP genotypes from Axiom chicken SNP arrays were established for samples with known H system serological types. Sources of DNA included two elite Hy-Line White Leghorn lines segregating for alloantigen H, non-pedigreed samples from the Northern Illinois University (NIU) DNA bank, plus inbred line samples. Sequence information was also available for the commercial and inbred lines. Results: GWAS results from the elite Hy-Line lines and NIU DNA bank samples showed a very strong peak in the same 4.20–4.30 Mbp region on chromosome 24. Predicted cell membrane expression and the presence of non-synonymous SNP were criteria to identify candidate genes. Seven genes in this region have membrane-associated products: MCAM (CD146), THY1, MFRP, CLDN25, KCNJ14L, ABCG4, and PDZD3. However, only MCAM had an SNP variation that matched the serological haplotypes. Lines known to be segregating for the H system had concordance rates between serological results and SNP haplotype of 95% for both the elite HYL lines and 99% for the NIU samples, indicating that the MCAM (CD146) gene encodes the chicken H blood system. Conclusions: The gene product is a cell adhesion molecule affecting multiple activities including angiogenesis, development, cell differentiation, cell migration, signaling transduction, and immune responses. Long, short, and soluble isoforms are found in chickens. The described DNA-based typing methods facilitate future investigations to examine H haplotype frequencies in lines with identified differential responses such as growth or immune responses. Determining H haplotype association with egg production, feed conversion, and other traits with economic importance will aid in determining the significance of this immune-related gene in overall poultry health.

## 1. Introduction

Chicken red blood cell alloantigens define genetic differences among individuals of that species. Landsteiner and Miller [1] were the first to study these alloantigens using antisera made by injecting chicken blood into rabbits. Ten different antisera were produced that, after adsorption, defined eight different blood types. Beginning in 1948, two different laboratories identified seven chicken alloantigen systems. Briles and co-workers found the A, B, C, D, and E systems [2,3,4,5]. The A and E systems showed close linkage, whereas the other three systems were not linked [3]. Independent studies by D. G. Gilmour [6,7,8] described four of the same systems (A, B, C, and E). His investigations also found two new alloantigen systems, designated L and N [6,7,8]. Therefore, chicken alloantigens A, B, C, D, E, L, and N [8,9] were known by 1960.

Two systems, F [9] and G [10], were next in order of discovery. However, comparison tests with existing antisera revealed that the F system was identical to the L system found by Gilmour [6]. The G system was likewise identical to the B blood group. Alloantigen H was the next unique system discovered. Multiple alloantisera produced against known systems were reactive against additional antigens. Adsorption of these sera removed extraneous reactivity, leaving sera that reacted to two alleles of a new system, named H. The alleles were designated H^1^ and H^2^ [9,11].

Bitgood and associates [12] crossed five different chromosomal rearrangement stocks to map genes, including pea comb (P), blue egg (O), and alloantigen systems A, E, H, and P. The paper reported that none of the alloantigen systems were associated with the short arm of chromosome 1. On the other hand, important linkage evidence was found for alloantigen systems. The data confirmed linkage between the A and E alloantigens at 1.3 cM and revealed that alloantigen H was linked to white skin (W+) at 17.2 cM. More recently, the dominant allele of beta-carotene dioxygenase 2 (BCDO2) [13,14], found on chromosome 24, has been identified as responsible for white skin. This finding suggests that the gene responsible for the chicken H blood system is located on chromosome 24.

Embryonic and post-hatch expression of multiple alloantigen systems were studied by Kopti and associates [15]. Indirect immunofluorescence tests showed that the H system was expressed on definitive erythrocytes instead of the primitive cell. Alloantigen H expression on erythrocytes at days 5, 6, and 7 of embryonic development was 0, 85, and 100%, respectively. One hundred percent expression of the H system continued from day 7 throughout the study, which concluded at 4 weeks of age.

Studies examining physiological effects of alloantigen H have been sparse. Erythrocyte alloantigen alleles were tested in the Storrs strain of hereditary muscular dystrophic (MD) chickens. Alloantigen H, fixed for the H^2^ allele, was one of five systems lacking segregation in a sample of 16 dystrophic chicks. A test backcross designed to produce both normal and affected progeny segregating for multiple alloantigen systems demonstrated no association between the MD incidence and any alloantigen system, including H [16].

The alloantigen H effect on immune responses was assessed by three different approaches. First, the allele frequencies of multiple alloantigen systems were measured in two lines divergently selected for the antibody response against sheep red blood cells (SRBCs). Birds from a common founder population were injected with a low dose (0.25% SRBC). The antibody titer 5 days post-injection was the selection criteria. After ten generations of selection, the high-antibody (HAS) line was fixed for the H^2^ allele, whereas the low-antibody (LAS) line had frequencies of 24% H^1^ and 76% H^2^ [17]. Three generations later, the frequencies were similar, with 25% H^1^ and 75% H^2^ [18]. The 25% H^1^ frequency suggested a contribution to lower antibody response.

Matings were used to generate progeny segregating for two MHC alleles and multiple alloantigen alleles. The first mating type produced B^19^B^19^ and B^19^B^21^ progeny that were tested at 2 and 4 weeks of age. Neither the amount of macrophage phagocytosis stimulated by *Escherichia coli* or the number of bacteria engulfed was affected by alloantigen H in 175 progeny tested at either age [19]. Another mating produced B^2^B^2^ or B^2^B^5^ genotypes that were segregated for multiple alloantigen systems. Alloantigen H did not affect macrophage nitrite or IL-6 levels in these birds [19,20].

The H system was examined for its effect on response to Rous sarcoma virus (RSV). Several matings using a single sire and multiple dams generated progeny segregating for combinations of multiple alloantigens, including the B system [21]. Progeny were injected with RSV, and the resulting tumor growth was measured. The tumor growth profile did not differ among H^1^H^1^, H^1^H^2^, or H^2^H^2^ genotypes in either B complex cohort, B^2^B^5^ or B^5^B^5^. Genotype H^1^H^1^ had tumor growth that was lower than that found in H^1^H^2^ or H^2^H^2^ genotypes in the B^2^B^5^ cohort, but the values did not attain significance [21].

The genes for six chicken alloantigen systems have been identified [20]. Tests for the different alleles of the A, B, D, E, I, and L systems have been developed [22,23,24,25,26]. The DNA techniques have accelerated identification and facilitated examination of alloantigen impacts on production traits. In this context, the objective of the current study was to find the chromosomal location and identify the gene responsible for chicken alloantigen H.

The candidate gene responsible for the H system is the melanoma cell adhesion molecule (MCAM, also known as CD146), a cell surface glycoprotein that was initially identified in humans as a melanoma tumor antigen [27]. It was recognized as a molecule that could identify severe lesions. Subsequently, it was found to be involved in multiple cellular processes through ligand binding, including T-cell receptor signaling, angiogenesis, and leukocyte transmigration [28,29]. The gene contains five immunoglobulin domains and is recognized as a member of the immunoglobulin superfamily (IgSF). It is found in two isoforms (long and short) due to alternative splicing of exon 15, as well as a soluble form found in plasma [29]. In chickens, the molecule was characterized as a hemopoietic cell adhesion molecule (HEMCAM) [30] for being expressed on embryonic bone marrow hematopoietic progenitors.

## 2. Materials and Methods

### 2.1. Genetic Material

DNA from birds with H system serological information was available from multiple unrelated sources, as listed in Table 1. Samples from the Northern Illinois University (NIU) DNA bank and two Hy-Line International (HYL), Dallas Center, IA, elite White Leghorn lines (WL1 and WL2) had been typed previously by Elwood and Ruth Briles at the NIU immunogenetics lab. Information on H system status for inbred lines was obtained from multiple sources. The NIU DNA bank consists of over 2500 samples, for which serological alleles for one or more blood systems had been previously determined. These samples came from a NIU population that was developed from multiple breeds, primarily Ancona and White Leghorn. This stock was used primarily for allele-specific alloantisera production, reactive against multiple chicken blood systems. Samples with H system allele typing information were identified and used for this study. The Hy-Line samples were from elite White Leghorn lines utilized to produce commercial egg production multi-line cross flocks. For two of these lines, H allele segregation was known for multiple generations. The HAS and LAS lines were initially derived from the Cornell random-bred WL [31] and have undergone more than 50 years of divergent selection for either high (HAS) or low (LAS) antibody levels following immunization with sheep red blood cells. H system information for both lines was previously reported [18], indicating that HAS was fixed for H^2^ and LAS was segregating for H^1^ and H^2^ in generations 10 and 13. The HAS and LAS samples we utilized were obtained from generation 42.

Lines UCD-001 (Red Jungle Fowl) and UCD-003 (WL) are inbred stocks developed at the University of California Davis (UCD) by inbreeding since 1956 [32]. UCD-001 is the line source that provided the DNA for the original chicken genome reference [34]. DNA was available from the sequenced reference individual, plus 10 additional samples from the same line collected in 2000. Samples from UCD-003 were obtained in 2004. Samples from the inbred line 15I_5_ [33] were obtained from ADOL in 2004.

### 2.2. GWAS Analysis Using SNP Genotypes and Individual DNA Samples

A total of 159 samples (52 from WL1 and 107 from NIU), for which H system serological data was known, were used for the genome-wide association study (GWAS) analysis. SNP genotype information was obtained for each sample using an AXIOM SNP chip (Affymetrix, Santa Clara, CA, USA) with 60,496 probes in total. The minimum call rate was required to be above 95% with a minor allele frequency of 0.05, resulting in 58,045 SNPs available for further analysis. GWAS was performed by regressing the number of H^1^ allele copies in individuals (coded as 0, 1, 2) on their SNP genotypes using assoc command in Plink. An association signal exceeding Bonferroni-corrected negative log10 (*p* value) of 6.065 was considered significant. GWAS results are presented as a Manhattan plot using ggplot2 v4.0.2 [35]. The same analysis was also performed separately for NIU and WL samples to verify that the signal was not a result of population structure.

### 2.3. SNP Selection and Genotyping

Genome sequence information was available from multiple HYL lines from a previous sequencing project [36], though H system segregation information was available for only two White Leghorn lines (WL1 and WL2). Genome sequences were visualized using GenomeBrowse 3.1.0 from GoldenHelix (Bozeman, MT, USA).

SNP for genotyping were identified using Build 6 (GRCg6a) alignment of genome sequences of the HYL lines. The analysis focused on the GWAS peak region, and SNP in this region were selected based on their segregation within the two HYL lines that were known to segregate for the H system. These SNP included synonymous, non-synonymous, and potential splice-site variants impacting SNP. The PACE^®^ (PCR Allelic Competitive Extension) chemistry (3CR Bioscience Ltd., Harlow, UK), which employs one common primer and two allele-specific primers with fluorescence detection of end-point reads, was used to identify SNP alleles [37]. Additional SNPs were included to further evaluate flanking region genes as potential candidates. Using the SNP genotyped within the candidate gene, gene haplotypes were identified. Where possible, the haplotype number assigned was consistent with the serological allele number, and additional haplotypes were numbered in order of their discovery.

### 2.4. Splice-Site Variant Analysis

Splice-impact screening of intronic variants in the MCAM (CD146) locus was performed using the same pipeline described previously for chicken blood system variant annotation [26]. SpliceAI v1.3.1 was used as the primary predictor of splice impact [38]. Orthogonal checks included ASSP [39], MaxEntScan [40], and RBPmap motif analysis [41].

### 2.5. Multiple Sequence Alignment and Topology Visualization

Chicken MCAM protein sequences were retrieved from NCBI RefSeq/GenBank (NP_001004768.1, BAA08648, BAA07563.1, NP_001382961.1, CAA70080, NP_001382962.1, CAA70081, and CAA70079) and aligned using MUSCLE [42]. For membrane topology visualization, representative full-length membrane isoforms corresponding to major haplotype and cytoplasmic tail classes were selected: long (NP_001004768.1) and short (BAA07563.1). Predicted topology schematics were generated using Protter [43] to annotate extracellular, transmembrane, and cytoplasmic regions in the architectural context of long versus short isoform. Predicted human MCAM long and short isoforms (XP_054224795.1 and XP_054224797.1, respectively) were included for reference.

### 2.6. Protein Structural Prediction and Pocket Identification

Representative extracellular domain isoforms of the selected long and short class described above were modeled using AlphaFold2 via ColabFold, with num_relax = 1 and template_mode = pdb100/auto [44,45]. Model boundaries were cross-checked by comparing predicted structures with DeepTMHMM v1.0.44 [46] annotations and visual inspection in PyMOL v3.1 [47]. For structural effect testing, in silico substitution was introduced into H sequences at the 482nd amino-acid position, and the same AlphaFold2/ColabFold workflow was applied to generate corresponding variant models.

Ligand-accessible pockets were identified using two independent approaches. CASTpFold was run using the default 1.4 Å probe radius to define solvent-accessible cavities; the dominant pocket for each model was selected based on cavity volume and surface vertex count [48]. In parallel, Fpocket v2 was run with default parameters to identify cavities as clusters of α-spheres derived from Voronoi tessellation and to compute cavity-level statistics including pocket score, druggability score (ligand-binding potential), volume, and hydrophobicity [49]. Finally, overlapping CASTpFold/Fpocket regions were mapped back onto structures in PyMOL.

### 2.7. Multi-Assembly Extraction and Gene Variant Annotation

To characterize MCAM sequence variation across chicken genome assemblies, genome FASTA files were downloaded using NCBI Datasets (*n* = 47 assemblies, spanning chromosome-, scaffold- and contig-level builds) (Table S1). The MCAM locus was defined on the Red Jungle Fowl reference genome (GRCg6a; chromosome 24: 4,240,683–4,248,590) and sequence information was extracted with samtools faidx [50] to serve as the query interval. For each assembly, the query interval was aligned to the assembly using minimap2 (-x asm5 -c) [51]. The top-scoring locus in each assembly was selected based on alignment identity and query coverage (≥80% identity and alignment length ≥500 bp), extracted from the assembly sequence, and they were oriented to match the reference strand (forward orientation). Loci from all assemblies were concatenated into a combined FASTA and aligned with MUSCLE to confirm locus concordance and to screen for gross misalignments or paralogous matches.

For variant discovery in reference coordinates, variants within the defined MCAM interval were called using bcftools by restricting mpileup to the interval followed by variant calling with call -mv [52]. Known variants from Ensembl/dbSNP (gallus_gallus_gca000002315v5.gvf) for the corresponding interval were retrieved and used to cross-check cohort variant identities and positions.

Human variants overlapping the MCAM locus were first identified by coordinate-based extraction from a GRCh38 chr11 whole-genome VCF spanning the MCAM interval (chr11:119,308,529–119,321,521; CCDG_14151_B01_GRM_WGS_2020-08-05). These candidate sites were then cross-referenced against the NCBI dbSNP GRCh38 VCF so that the plotted set reflected dbSNP-represented variants at the MCAM locus and allowed allele/position concordance checks. Predicted molecular consequences were assigned relative to Ensembl GRCh38.112 transcript models (VEP/Ensembl consequence terms). For consistency in exon/CDS architecture and amino-acid mapping, a single MCAM transcript (ENST00000264036) was used as the reference plotting model for exon/CDS coordinates.

For visualization, chicken and human variants were grouped into four functional classes based on consequence: non-synonymous (missense, frameshift, start/stop gained or lost), synonymous, splice-related (splice donor/acceptor and other splice consequences), and noncoding/modifier (all remaining consequences, including UTR and intronic annotations). The chicken candidate snp at AA 482, as well as human-regional missense variants within AA 495–548 and those with hydrophobic substitutions (A, V, I, L, M, F, W, Y), were noted. Human variants were further binned (50 bp), set to the median genomic x-coordinate of variants in that stratum, and stem height-transformed (log1p to max observed; gamma compression) for visual clarity.

## 3. Results

### 3.1. GWAS

**Figure 1:**
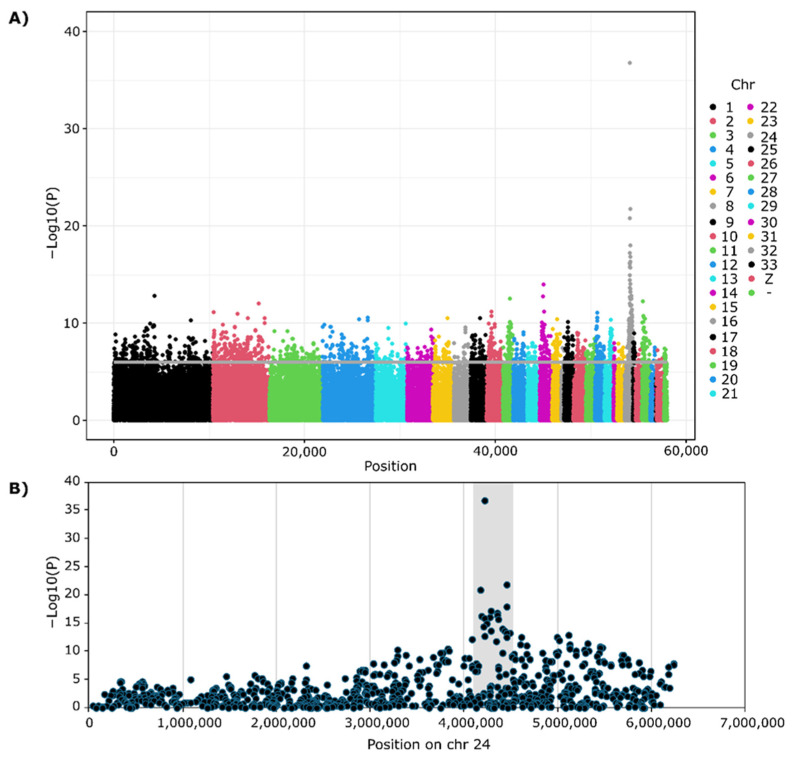
Genome-wide association analysis of HYL and NIU samples. (**A**) Manhattan plot showing results from a GWAS using 54K SNP genotypes with HYL and NIU data combined. Each point represents a SNP plotted by genomic position (*x*-axis) and −log10(P) (*y*-axis), with chromosomes ordered left to right and distinguished by alternating colors. The horizontal gray line indicates the genome-wide significance threshold. (**B**) Regional association plot for chromosome 24 highlighting the major association peak centered at 4,218,809 bp on chr 24 (shaded area), corresponding to the region containing MCAM. SNPs are plotted by chromosomal position and −log10(P), illustrating the local signal underlying the genome-wide peak.

The GWAS analysis showed a single peak on chromosome 24 (Figure 1A), between 4.17 and 4.45 Mbp, thus confirming previous reports locating the H system on chromosome 24. This region contains 7 genes with membrane-associated products: MCAM (CD146), THY1, MFRP, CLDN25, KCNJ14L, ABCG4, and PDZD3. This same region was found for both the NIU and HYL samples, thus confirming the region for further study. For all samples combined, the strongest SNP (−log10 = 36.8) was at positions 4,218,809 (rs315704849), located in USPS2 with the next two strongest SNPs (−log10 = 20.8 rs316105325; 1log10 = 21.8 rs316105325) located at 4,178,411 and 44,452,826, thus setting the boundaries for a focused study. Figure 1B shows a zoom view of this region.

### 3.2. SNP Genotyping

Genome sequences centered at 4,218,908 bp on chromosome 24 were examined to identify those SNPs that were consistent with known segregation patterns within the HYL lines. A total of 30 SNPs were identified (from 4,178,411 to 4,452,826) to encompass the GWAS region. Close examination of the individual SNP genotype results showed that only those SNPs within MCAM (CD146) fit the expected segregation patterns. The additional SNP in the flanking regions confirmed the exclusion of other genes, thus identifying MCAM (CD146) as the candidate gene.

### 3.3. MCAM (CD146) Haplotypes vs. Serology

Focus was placed on the MCAM gene from which 20 SNP were identified, genotyped and utilized for MCAM haplotype identification. Table 2 shows these 20 SNPs, including their rs numbers (where available), genomic and gene locations, plus the type of change they are predicted to produce. The 20 SNPs located within the MCAM gene that were used to define the MCAM haplotypes are numbered (11–30) for ease of distinction. Fourteen MCAM haplotypes were identified, with the H system serological allele classified for 10 of the haplotypes.

MCAM-H01 and MCAM-H03 were found for the serological H^1^ allele, whereas other haplotypes are found for the serological H^2^ allele. Haplotype MCAM-H08 was identified from the reference genome sequence information (UCD-001) and matches the haplotype SNP typed from the reference DNA sample. Genotyping of the additional samples from line UCD-001 revealed the segregation of two haplotypes, MCAM-H08 and MCAM-H09. The associated H system serological allele is unknown for line UCD-001 and thus a serological allele cannot be assigned to these two haplotypes. Additional haplotypes found are included for completeness even though the serological H allele may be unknown. Common haplotypes are found between lines, as seen for MCAM-H01 being in WL1, WL2, and NIU, with all identified as being associated with the serological H^1^ allele, and MCAM-H02 being found in WL1, NIU, and UCD-003 with the serological H^2^ allele. It is interesting to note that the UCD-001 samples showed 2 haplotypes, MCAM-H08 (identical to the reference sequence) and MCAM-H09, which differs from MCAM-H08 by four SNP, one of which (SNP 19) results in a non-synonymous change (Q234R). The inbred line UCD-003 (serologically H^2^) contained two haplotypes (MCAM-H02 and MCAM-H14), which differ by one synonymous SNP (SNP 18) that is not likely to impact protein structure. The observation of the same MCAM haplotypes associated with the same H system serological alleles in unrelated lines adds confidence to the identification of MCAM as the gene product responsible for the chicken H blood system.

Consistency is seen for all MCAM haplotypes encoding the H^2^ allele for SNP number 26 (rs739155076), which shows a non-synonymous difference of V482I between all H^2^ vs. H^1^ serological alleles. This finding will be discussed in further detail below. This difference suggests that this single amino-acid change is responsible for the antigenic differentiation between the two H system alleles.

### 3.4. Serological Discrepancies

The concordance between serological allele and MCAM haplotypes is less than 100%. Within the two HYL lines, there was 89.2 and 95.0% concordance (WL1 and WL2, respectively), whereas the concordance within the NIU samples was 99%. Within these errors, there were six individuals that showed MCAM haplotype homozygosity, though the serology indicated they were heterozygous. Examination of pedigree information showed that the sire MCAM haplotype was homozygous, with a haplotype inconsistent with their progeny serology, but consistent with their DNA-derived MCAM haplotype, confirming serological misidentification for the progeny. Thus, all six samples that could be checked through their pedigree confirmed serological mistyping. Similar examples of low-level serological mistyping have been found with other chicken blood systems [22,23,24,25].

### 3.5. Number of H System Serological Alleles

A minor discrepancy exists in documentation of the alloantigen H alleles. Two original publications cited two alleles, H^1^ and H^2^ [9,11]. Later, a single publication by Briles and Gilmour [53] reported three alloantigen H alleles. No subsequent work defined the third H allele. In fact, Briles [54] stated that the H system had two alleles. Studies using segregating haplotype combinations of multiple alloantigens included only H^1^ and H^2^. This inconsistency may be explained by three alternative views. First, the H allele number may have been misstated [53]. Next, the third H allele was later found to be synonymous with either H^1^ or H^2^, but that finding was not published. Finally, the H^3^ allele was very rare such that it was not reported after 1979. We are proceeding with the designation of two H alleles without supporting any of these explanations.

### 3.6. H^1^ and H^2^ Isoforms Are Characterized and Classified

Eight annotated chicken MCAM protein isoforms were retrieved from public databases and aligned to evaluate haplotype background and shared sequence features (Figure S1). Based on coding variation at position 482 (p.Val482Ile; rs739155076), sequences were segregated into two groups: three isoforms encoded as Val482 (designated H^1^-type), and five encoded as Ile482 (designated H^2^-type). One H^2^-type record represented a truncated soluble form lacking full-membrane architecture and was therefore not considered the primary structural candidate for blood-type determination. In this isoform, retention of an intronic segment downstream of the V482I site (chr24:4,246,227–4,246,286, GRCg6a) introduces a premature termination codon, resulting in loss of the cytoplasmic tail.

Public transcript annotations describe additional 5′ coding differences between Val482- and Ile482-containing sequences, including p.Pro37Leu (rs738076238), p.Arg69His (rs313899164), and a reported indel-associated frameshift affecting residues 43–89 (H^1^: chr24:g.4244285delC; H2: chr24:g.4244108delC) [30]. Several of these upstream substitutions showed partial concordance with the V482I background, suggesting conditional or context-dependent linkage across the 5′ region. However, segregation analysis in our genotyped populations and follow-up PCR did not support consistent linkage across this 5′ interval, and the reported indel region was not identified in our assembly cohort. Because these upstream variants did not define a stable or clearly bounded haplotype block, they were not usable to classify H^1^- and H^2^-type sequences. In contrast, p.Val482Ile segregated cleanly across datasets (see Table 2) and was retained as the defining coding marker associated with serologic differences and distinguishing H^1^ and H^2^ alleles.

Comparison with annotated human isoforms demonstrated conserved overall protein topology, including variability in membrane-proximal extracellular segments (Figures S2 and S3). Among nine human isoforms examined, long and short tail forms exhibited 21- or 57-amino-acid contiguous differences in the membrane-proximal ectodomain due to indels in exons 12–13. This indicates that localized sequence variability in this region is compatible with normal CD146 isoform architecture and supports the interpretation that the chicken H-associated site lies within a structurally variable yet evolutionarily conserved domain context.

### 3.7. Differential Splicing Affects Tail Length

Both H^1^- and H^2^-type protein sequences included long and short cytoplasmic tail isoforms (Figure S3). We therefore examined whether splice-associated variants could account for this tail-length diversity. Among intronic variants flanking the tail-defining exon junctions, rs733367385 (chr24:4,246,100 T>C), located upstream of exon 11, emerged as the strongest splice candidate in both our SNP panel and segregation analyses (Table 2). A second nearby intronic variant, rs730957023 (chr24:4,247,573 T>C), located upstream of exon 14, showed weaker but directionally consistent in silico splice predictions (Figure S3).

SpliceAI v1.3.1 predicted acceptor-gain effects at both loci, with a larger shift at rs733367385 (DS_AG = 0.23), numerically exceeding neighboring variants (≤0.05), whereas rs730957023 showed only a modest signal (DS_AG = 0.02). MaxEntScan supported strengthening of the 3′ acceptor motif at rs733367385 (Δ +0.65) and indicated a smaller acceptor shift at rs730957023 (Δ +0.86 from a lower baseline score). MaxEntScan supported strengthened 3′ acceptor motifs, and RBPmap indicated loss of predicted PUF60 binding motif at both loci, consistent with altered 3′ splice-site selection underlying the observed tail-length differences. Mechanistically, this is consistent with the known CD146 tail-length switch in humans, where exon 15 skipping introduces a premature stop codon, producing a shorter cytoplasmic tail isoform.

The MCAM haplotypes defined in Table 2 do not show any variation within SNP rs733367385 (chr24:4,246,100 T>C) suggesting a lack of the short cytoplasmic tail isoforms within the samples used. However, sequence information from other Hy-Line elite lines show the presence of this alternate-splice SNP, indicating that short isoforms are likely found within these lines. Additional MCAM genotype information is not available in these other lines as they did not have any H system serological information and were not MCAM SNP genotyped.

### 3.8. Haplotype-Associated Differences in Predicted Ectodomain Pocket Architecture

**Figure 2:**
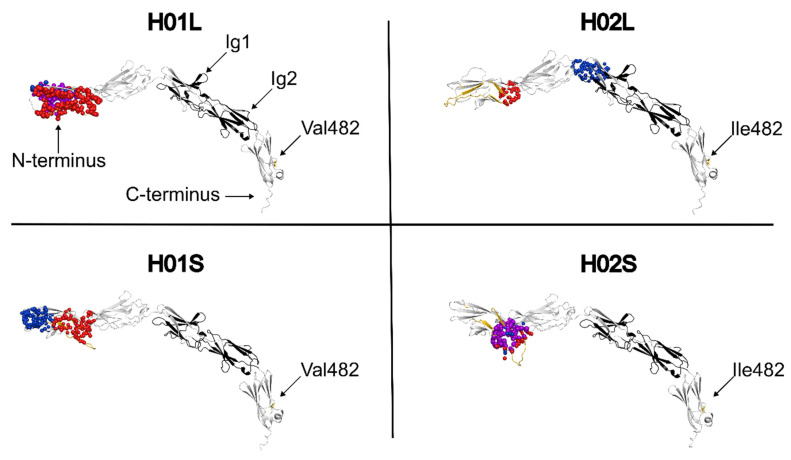
Comparison of predicted ligand-accessible pockets between chicken H^1^ and H^2^ ectodomain isoforms. Pocket regions identified by CASTpFold (red) and Fpocket (blue) are shown, with overlapping predictions indicated in purple. In H^1^ isoforms, the dominant pocket localizes to the distal N-terminal region, whereas in H^2^ isoforms, the pocket shifts toward the Ig1 domain. Ig1 and Ig2 domains are shown in black, the N-terminal isoform-variable region (residues 37–89) is shown in yellow, and residue 482 is indicated. Ectodomains are oriented with the N-terminus to the left and the C-terminus to the right.

Protein modeling of the MCAM protein was performed to better understand the potential impact of the identified serological epitope distinguishing H^1^ and H^2^ alleles. Structural modeling of the MCAM ectodomain revealed a redistribution of predicted ligand-accessible cavities between protein isoforms encoded by MCAM-H01 and MCAM-H02 haplotypes (Figure 2), as well as between the long and short cytoplasmic tail isoforms within each haplotype. Structural modeling of the MCAM ectodomains revealed a consistent redistribution of predicted ligand-accessible cavities between H01 and H02 isoforms (Figure 2) and between the long and short isoforms within H01 and H02. In the H01 long isoform (H01L), both CASTpFold and fpocket identified the dominant pocket at the distal N-terminal end of the ectodomain, spatially separated from the Ig1/Ig2 modules. In contrast, in the H02 long isoform (H02L), the highest-scoring cavity localized closer to the Ig1 region rather than at the extreme N-terminus. The Ig-associated regions form part of the structurally conserved core, whereas the distal N-terminal segment exhibits greater positional variability in the models.

Structural comparison of H01L models indicated that residues ~50–85 form a flexible N-terminal segment that follows distinct spatial arcs relative to the Ig core. This region comprises a substantial portion of the predicted distal pocket surface and overlaps the putative variable region (Figure S1), linking variation in its placement to differences in cavity architecture. In both H02 isoforms, relocation of the dominant pocket toward the Ig1–Ig2 region was accompanied by reduced ligand-binding potential (drug score) and decreased surface hydrophobicity compared with H01. Quantitatively, the H01L N-terminal pocket exhibited greater predicted volume and ligand-binding potential (drug score) than the H02L Ig1-associated pocket (Fpocket pocket score 41.4 vs. 31.2; real volume ~2600 vs. ~1260 Å^3^; drug score 0.80 vs. 0.42), a pattern also observed in the short isoforms (H01S, H02S). Together, these findings indicate a haplotype-associated shift in pocket localization and cavity properties.

### 3.9. Hydrophobic Missense Changes Are Shared in Chickens and Humans

**Figure 3:**
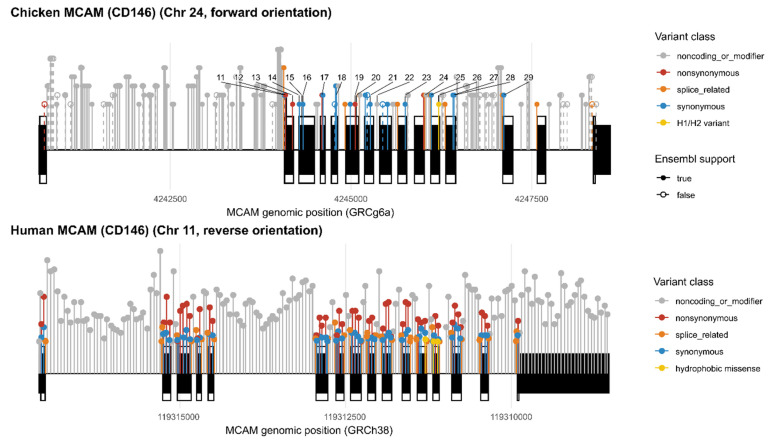
Distribution of sequence variants across the MCAM (CD146) locus in chickens and humans. Variants are plotted relative to a reference transcript model and colored by consequence class (non-synonymous, synonymous, splice-related, noncoding/modifier). The chicken H-associated site (p.Val482Ile) is indicated, and human missense variants in the membrane-proximal ectodomain region are shown. Human variants are binned for density, and stem heights are scaled for clarity.

Chicken variant annotation across 235 polymorphic sites in the MCAM interval identified predominantly noncoding variation (200/235 intronic/UTR), with comparatively few coding-impacting sites (18 synonymous and 7 missense) and a small set of splice-region/polypyrimidine-tract variants (10) (Figure 3). All seven protein-altering variants were annotated as missense and were strongly skewed toward hydrophobic substitutions: 6/7 either introduced a hydrophobic residue (C22W, P37L, T156I) or represented conservative hydrophobic exchanges (V70I, V482I), while only one missense variant (Q244R) did not involve a hydrophobic residue.

Notably, the serology-associated site V482I is one of only two conservative hydrophobic swaps in the dataset and lies in the membrane-proximal ectodomain region highlighted in the structural analyses (Figure 3). A similar pattern is observed in human MCAM, where consequence-annotated variants are predominantly noncoding, and synonymous substitutions outnumber most individual classes of coding change. Among missense variants, hydrophobic residues are frequently involved, including conservative aliphatic substitutions within the membrane-proximal ectodomain, (eg. I511V, L512V, V517I, and L522V). Thus, enrichment of hydrophobic amino-acid exchange in this region appears conserved across species.

Public mRNA records annotate additional 5′ differences between the Val482 and Ile482 transcript sets (e.g., p.Pro37Leu; rs38076238), but in our genotyped birds, these upstream variants showed incomplete co-segregation with the serological H allele-defining V482I marker (Figures S1 and S4–S6). For example, rs38076238 co-tracked with V482I in 94% of H^1^-class individuals (33/35) but only 50% of H^2^-class individuals (4/8), indicating inconsistent linkage across populations. The single-base indel differences reported in some transcript records were also not detected in our DNA-based assays. These findings support V482I as the most consistent coding marker distinguishing H^1^- and H^2^-serological alleles in this study, but testing would be necessary for confirmation.

## 4. Discussion

The GWAS analysis used a small set (60K) of SNPs, which successfully identified the region of interest containing the candidate gene. However, this SNP subset did not contain segregating SNPs with the candidate gene. Individual genotyping of additional SNP within this region identified the candidate gene and helped to eliminate other genes within the region. A subset of H system serologically defined samples was used for the GWAS analysis. The remaining samples allowed independent confirmation of the candidate gene. Also, additional independent confirmation of the candidate gene was obtained utilizing samples that were from a completely independent line. Information from several inbred lines showed consistency of MCAM haplotypes, providing further confirmation of MCAM as the candidate gene.

Single exofacial amino-acid substitutions frequently underlie clinically significant red blood cell antigens, and their immunogenicity depends strongly on structural context, including surface accessibility and local fold geometry [55]. While V482I represents a conservative hydrophobic substitution, it could contribute to H^1^/H^2^ serologic differentiation either directly, by altering a conformational epitope, or indirectly, by modifying the local extracellular environment within a haplotype-defined sequence background. This possibility can be empirically tested through site-directed mutagenesis and functional serologic assays. Even subtle side-chain alterations can shift epitope presentation sufficiently to affect antibody recognition.

The MCAM gene is conserved across vertebrates and retains the same overall architecture (signal peptide, multiple Ig-like domains, a transmembrane segment, and a cytoplasmic tail), but it shows moderate sequence divergence across species. In mammals, mouse MCAM is highly similar to human MCAM: the coding sequence is ~80.6% identical and the predicted protein is ~76.2% identical at the amino-acid level [56]. Conservation drops outside of the mammalian lineage; for example, zebrafish MCAM shares only ~30% overall amino-acid identity with mammalian/avian orthologs, consistent with preserved core features but has substantial divergence over evolutionary distance [57]. In chickens, the predominance of synonymous/noncoding variation over non-synonymous at MCAM fits with strong functional constraint on this receptor in closed, selected populations, and contrasts with the broader missense catalog observed at the human locus.

Assignment of the classical H blood system to MCAM/CD146 therefore reframes this receptor from a broadly described adhesion molecule to an erythrocyte phenotype. This dual identity aligns with the behavior of other immunoglobulin superfamily members that function both in tissue biology and as red cell antigens (e.g., basal cell adhesion molecule). Though MCAM was initially identified as a melanoma cell adhesion molecule, it has since been determined to have an active role in multiple cellular processes, including cell adhesion, migration, and signaling. It acts as a cell surface receptor, binding multiple ligands involving angiogenesis, inflammation, and immune responses [58]. The findings expand the functional context of MCAM and establish a mechanistic basis for its role in erythrocyte serology.

The limited data on alloantigen H effects on production or health-related traits offers opportunities to study the potential receptor, adhesion molecule, and erythrocyte phenotype functions. The development of a SNP-based typing system facilitates assessment of haplotype frequencies in highly selected stock. Alteration of alloantigen H frequencies in lines differing in egg production, growth, or disease resistance would suggest the H system affects the character under selection. Specific matings can be made to test such hypotheses. In addition, specific designed matings can produce progeny segregating for H system alleles. Any H system effects on production traits can be enhanced through selection, thereby improving performance.

## Figures and Tables

**Table 1:** Source of genetic material, number of samples genotyped on MCAM SNP panel, and serological H system allele previously reported present in the line.

Source	No.	H Alleles	References
NIU	120	H^1^, H^2^	Briles, unpublished
WL1	120	H^1^, H^2^	Briles, unpublished
WL2	139	H^1^, H^2^	Briles, unpublished
UCD-001	11	unk	none
UCD-003	27	H^2^	[32]
15I_5_	32	H^2^	[33]
HAS	28	H^2^	[17,18]
LAS	32	H^1^, H^2^ *	[17,18]

unk = unknown. * segregation based on generations 10 and 13.

**Table 2:** MCAM SNP, their rs numbers, and genome location.

RS ID	SNP Location	Ref/Alt	Location	Ref/Alt	SNP No.	Ref (Seq) #	H01	H02	H03	H04	H05	H06	H07	H08	H09	H10	H11	H12	H13	H14
rs738076238	4,244,094	C/T	exon 2	P37L	11	C *	T	C	T	C	C	C	T	C	C	C	C	C	C	C
n/a	4,244,107	-/C	exon 2		12	-	-	-	-	-	-	-	-	-	-	-	-	-	-	-
rs313899164	4,244,192	G/A	exon 3	R69H	13	G	A	A	A	G	G	G	G	G	G	A	A	A	A	A
n/a	4,244,284	-/C	exon 4	R77P	14	-	-	-	-	-	-	-	-	-	-	-	-	-	-	-
n/a	4,244,320	C/-	exon 4		15	C	C	C	C	C	C	C	C	C	C	C	C	C	C	C
rs1059525718	4,244,336	C/G	exon 5	R93R	16	C	C	C	C	C	C	C	C	C	C	C	C	C	C	C
rs314413736	4,244,599	C/T	exon 6	T155I	17	C	T	T	T	C	T	C	C	C	C	T	T	T	T	T
rs737441055	4,244,797	G/A	exon 7	P193P	18	G	G	G	G	G	G	G	G	G	G	G	G	G	G	A
rs732980879	4,245,058	A/G	exon 8	Q243R	19	A	A	A	A	A	A	A	A	A	G	A	A	A	A	A
rs733638595	4,245,074	C/A	exon 8	T248T	20	C	C	C	C	C	C	C	C	C	A	C	C	C	C	C
rs15222017	4,245,267	T/C	exon 9	C286C	21	T	C	T	T	C	T	C	C	T	C	T	C	T	T	T
rs15222021	4,245,361	C/T	intron 9		22	C	T	C	T	T	C	C	C	C	C	C	T	T	C	C
rs731472380	4,245,750	T/C	exon 11	D390D	23	T	C	C	C	C	T	C	C	T	C	T	C	C	T	C
rs733227834	4,246,024	T/C	intron 12		24	T	C	C	C	T	C	C	C	T	T	T	C	T	T	C
rs733367385	4,246,100	T/C	intron 12		25	T (LONG)	T	T	T	T	T	T	T	T	T	T	T	T	T	T
rs739155076	4,246,215	G/A	exon 13	V482I	26	G	A	G	A	G	G	G	G	G	G	G	G	G	G	G
rs314156118	4,246,412	C/T	exon 14	N517N	27	C	C	T	C	C	C	T	T	C	C	C	C	C	C	T
rs15222024	4,246,427	T/C	exon 14	S522S	28	T	C	C	C	C	C	C	C	T	T	C	C	C	C	C
rs737365405	4,247,115	G/A	exon 15	S534S	29	G	G	G	G	G	G	G	G	G	G	G	G	G	G	G
rs730957023	4,247,573	T/C	intron 15		30	T (LONG)	C	C	C	C	C	C	C	T	T	C	C	C	C	C
							H^1^	H^2^	H^1^	H^2^	H^2^	H^2^	H^2^	?	?	H^2^	H^2^	?	?	H^2^
							WL1	WL1	WL1	WL1	WL2	NIU	NIU	UCD-001	UCD-001	HAS	HAS	LAS	LAS	UCD-003
							WL2	NIU		NIU	NIU									
							NIU	UCD-003			15I_5_									

# identified based on reference sequence. * C, T, G, A = nitrogenous base found at a given position cytosine (C), thymine (T), guanine (G), or adenine (A).

## Data Availability

The original contributions presented in this study are included in the article and Supplementary Materials. Publicly available genome assemblies and variant datasets were obtained from NCBI and Ensembl as described in the Methods section. The data presented in this study are available on request from the corresponding author. Restrictions apply to the availability of these data due to proprietary content from Hy-Line International.

## Supplementary Materials

The following supporting information can be downloaded at https://www.mdpi.com/article/10.3390/genes17040412/s1. Figure S1: Alignment of chicken MCAM (CD146) protein isoforms; Figure S2: Alignment of human MCAM (CD146) protein isoforms; Figure S3. Membrane topology of chicken MCAM (CD146) haplotypes and splice isoforms compared with human isoforms; Figure S4. Exon 2 alignment of chicken MCAM (CD146) highlighting the P37L variant (rs38076238); Figure S5: Exon 3 alignment of chicken MCAM (CD146) highlighting the P70H variant (rs313899164); Figure S6. Chicken MCAM (CD146) alignment highlighting the V482I variant (rs739155076). Table S1. Metadata for 47 chicken genome assemblies used in MCAM (CD146) locus analysis, including breed, ecotype/isolate, assembly level, and locus extraction status.
