# Supplementary material for: MCAM (CD146) Gene Encodes Chicken Blood Alloantigen System H
Source: Genes (Basel). 2026 Mar 31;17(4):412. doi: 10.3390/genes17040412 (PMC13116496; doi:10.3390/genes17040412)
Supplement: Supplementary file 1 [file genes-17-00412-s001.zip › genes-4213633-supplementary.pdf]

Supplementary Information

Supplemental Tables

Supplementary Table S1. Metadata for 47 chicken genome assemblies used in MCAM (CD146) locus analysis, including breed, ecotype/isolate, assembly level, and locus extraction status.

Supplemental Figures

Supplementary Figure S1. Alignment of chicken MCAM (CD146) protein isoforms.

Supplementary Figure S2. Alignment of human MCAM (CD146) protein isoforms.

Supplementary Figure S3. Membrane topology of chicken MCAM (CD146) haplotypes and splice isoforms compared with human isoforms.

Supplementary Figure S4. Exon 2 alignment of chicken MCAM (CD146) highlighting the P37L variant (rs38076238).

Supplementary Figure S5. Exon 3 alignment of chicken MCAM (CD146) highlighting the P70H variant (rs313899164).

Supplementary Figure S6. Chicken MCAM (CD146) alignment highlighting the V482I 18 variant (rs739155076).

Supplementary Table S1: Metadata for 47 chicken genome assemblies used in MCAM (CD146) locus analysis, 22 including breed, ecotype/isolate, assembly level, and locus extraction status.

Assembly acc.	Assembly name	Breed (ectotype; isolate)	Level	Aligned	Locus len.
GCA_000002315.5	GRCg6a	Red Jungle fowl (isolate: RJF #256; inbred line UCD001)	Chromosome	Yes	7908
GCA_002798355.1	Ogye1.0	Yeonsan Ogye	Chromosome	Yes	7911
GCA_016699485.1	bGalGal1.mat.broiler.GRCg7b	Cross of Broiler mother + white leghorn layer father (isolate: bGalGal1)	Chromosome	Yes	7877
GCA_016700215.2	bGalGal1.pat.whiteleghornlayer.GRCg7w_WZ	Cross of Broiler mother + white leghorn layer father (isolate: bGalGal1)	Chromosome	Yes	7882
GCA_024206055.2	GGswu	Huxu	Chromosome	Yes	7891
GCA_024652985.1	ASM2465298v1	Rhode Island Red (isolate: Rhode_Island_Red_3_LI)	Chromosome	Yes	7898
GCA_024652995.1	ASM2465299v1	White Leghorn (isolate: White_Leghorn_3_WL)	Chromosome	Yes	7894
GCA_024653025.1	ASM2465302v1	Silkie (isolate: Silkies_3_SK)	Chromosome	Yes	7502
GCA_024653035.1	ASM2465303v1	Cornish (isolate: Cornish_3_KN)	Chromosome	Yes	7907
GCA_024653045.1	ASM2465304v1	Houdan (isolate: Houdan_3_HD)	Chromosome	Yes	7917
GCA_024679325.1	ASM2467932v1	Silkie (isolate: Silkies_2_wuji)	Scaffold	Yes	7913
GCA_024679355.1	ASM2467935v1	Daweishan (isolate: Daweishan_2_m5-6)	Scaffold	Yes	7879
GCA_024679375.1	ASM2467937v1	Liyang (isolate: Liyang_2_suyang)	Scaffold	Yes	7847
GCA_024679395.1	ASM2467939v1	Tibetan chicken (isolate: Tibetan_chicken_2_zangji)	Scaffold	Yes	7707
GCA_024679415.1	ASM2467941v1	White Plymouth Rock (isolate: White_Plymouth_Rock_2_y24)	Scaffold	Yes	7700
GCA_024679765.1	ASM2467976v1	Rhode Island Red (isolate: Rhode_Island_Red_2_luodao)	Scaffold	Yes	7779
GCA_024679905.1	ASM2467990v1	White Leghorn (isolate: White_Leghorn_2_laihang)	Scaffold	Yes	7880
GCA_024686275.1	ASM2468627v1	Fayoumi (isolate: Fayoumi_3_FYI)	Contig	Yes	7908
GCA_024686285.1	ASM2468628v1	Thailand Gamefowl (isolate: Thailand_Gamefowl_3_FTCB)	Scaffold	Yes	7752

GCA_024686295.1	ASM2468629v1	Chahua (isolate: Chahua_2_chahua)	Scaffold	Yes	7899
GCA_024686315.1	ASM2468631v1	Langshan (isolate: Langshan_2_langshan)	Scaffold	Yes	7885
GCA_024686355.1	ASM2468635v1	Asil (isolate: Asil_3_101A)	Scaffold	Yes	7893
GCA_024686465.1	ASM2468646v1	Naked Neck (isolate: Naked_Neck_3_201A)	Scaffold	Yes	7899
GCA_024687005.1	ASM2468700v1	Cornish (isolate: Cornish_2_am30)	Scaffold	Yes	7795
GCA_025370635.1	ASM2537063v1	Tibetan chicken (isolate: Tibetan_chicken_3_TB)	Chromosome	Yes	7898
GCA_027408205.1	bGalGal5.alt	Cobb (isolate: bGalGal5)	Scaffold	Yes	7910
GCA_027408225.1	bGalGal5.pri	Cobb (isolate: bGalGal5)	Chromosome	Yes	7913
GCA_027408255.1	bGalGal4.alt	Ross (isolate: bGalGal4)	Scaffold	Partial	1943
GCA_027557775.1	bGalGal4.pri	Ross (isolate: bGalGal4)	Chromosome	Yes	7844
GCA_030849555.2	ASM3084955v2	Daweishan (isolate: F025)	Chromosome	Yes	7906
GCA_030914265.2	ASM3091426v2	Piao (isolate: P17)	Chromosome	Yes	7892
GCA_030914275.2	ASM3091427v2	Wuding (isolate: W17)	Chromosome	Yes	7883
GCA_030979905.2	ASM3097990v2	Hu (isolate: H3)	Chromosome	Yes	7897
GCA_033088195.2	CAU_Silkie_2.0	Silkie (isolate: CAU_Silkie)	Chromosome	Yes	7917
GCA_034509865.1	ASM3450986v1	Silkie	Contig	Yes	6761
GCA_034509885.1	ASM3450988v1	Lueyang	Contig	Yes	6427
GCA_034769225.1	CVASU_Gallus_Gallus_GG1M	Bangladeshi Hilly Chicken (isolate: GG1M)	Chromosome	Yes	7887
GCA_034769275.1	CVASU_Gallus_Gallus_GG1F	Bangladeshi Hilly Chicken (isolate: GG1F)	Chromosome	Yes	7896
GCA_039997075.1	ASM3999707v1	Ginkkoridak (isolate: KLT)	Chromosome	Yes	7907
GCA_040436645.1	ASM4043664v1	Wenchang (ecotype: Hainan; isolate: WChapref)	Chromosome	Yes	7910
GCA_040436655.1	ASM4043665v1	Wenchang (ecotype: Hainan; isolate: WChapref)	Chromosome	Yes	7902
GCA_041920315.1	ASM4192031v1	Game fowl (isolate: XGF-202401-001)	Chromosome	Yes	7905
GCA_046463955.1	ASM4646395v1	Sulawesi Red Junglefowl (ecotype: Indonesia; isolate: SLWX2)	Contig	Yes	7919
GCA_052054655.1	ASM5205465v1	Unspecified chicken (isolate: SHJ)	Chromosome	Yes	7238
GCA_034509845.1	ASM3450984v1	Hailanhe	Contig	No	NA

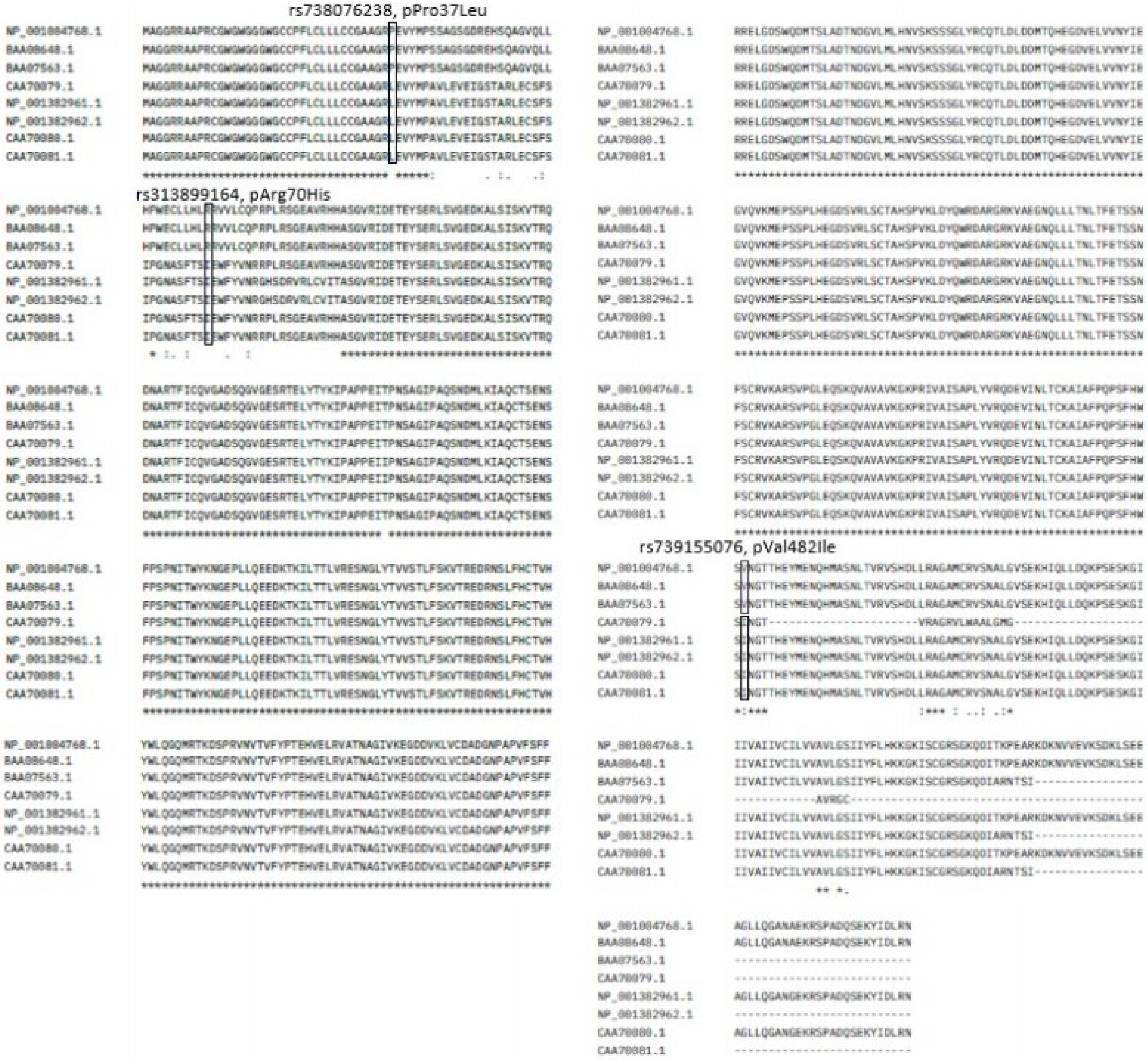

Supplementary Figure S1: Alignment of chicken MCAM (CD146) protein isoforms. Conserved residues are indicated as fully conserved (*), strongly conserved (:), weakly 27 conserved (.), or non-conserved. Variant positions Pro37Leu, Arg70His, and Val482Ile 28 are highlighted. Putative N-terminal variable region: 37-89.

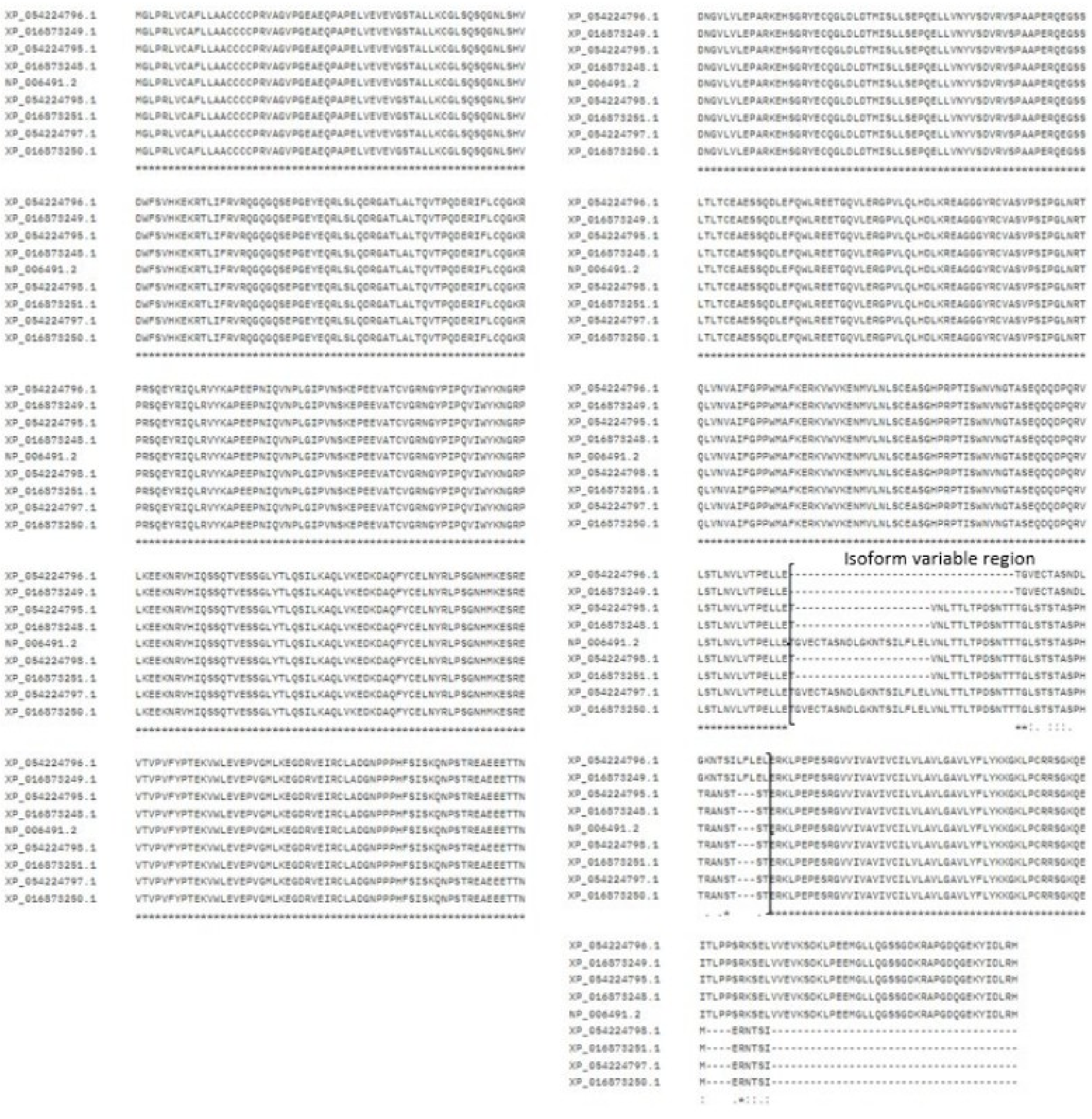

Supplementary Figure S2: Alignment of human MCAM (CD146) protein isoforms. Conserved residues are indicated as fully conserved (*), strongly conserved (:), weakly conserved (.), or non-conserved. The C-terminal isoform-variable region distinguishing long and short forms is indicated.

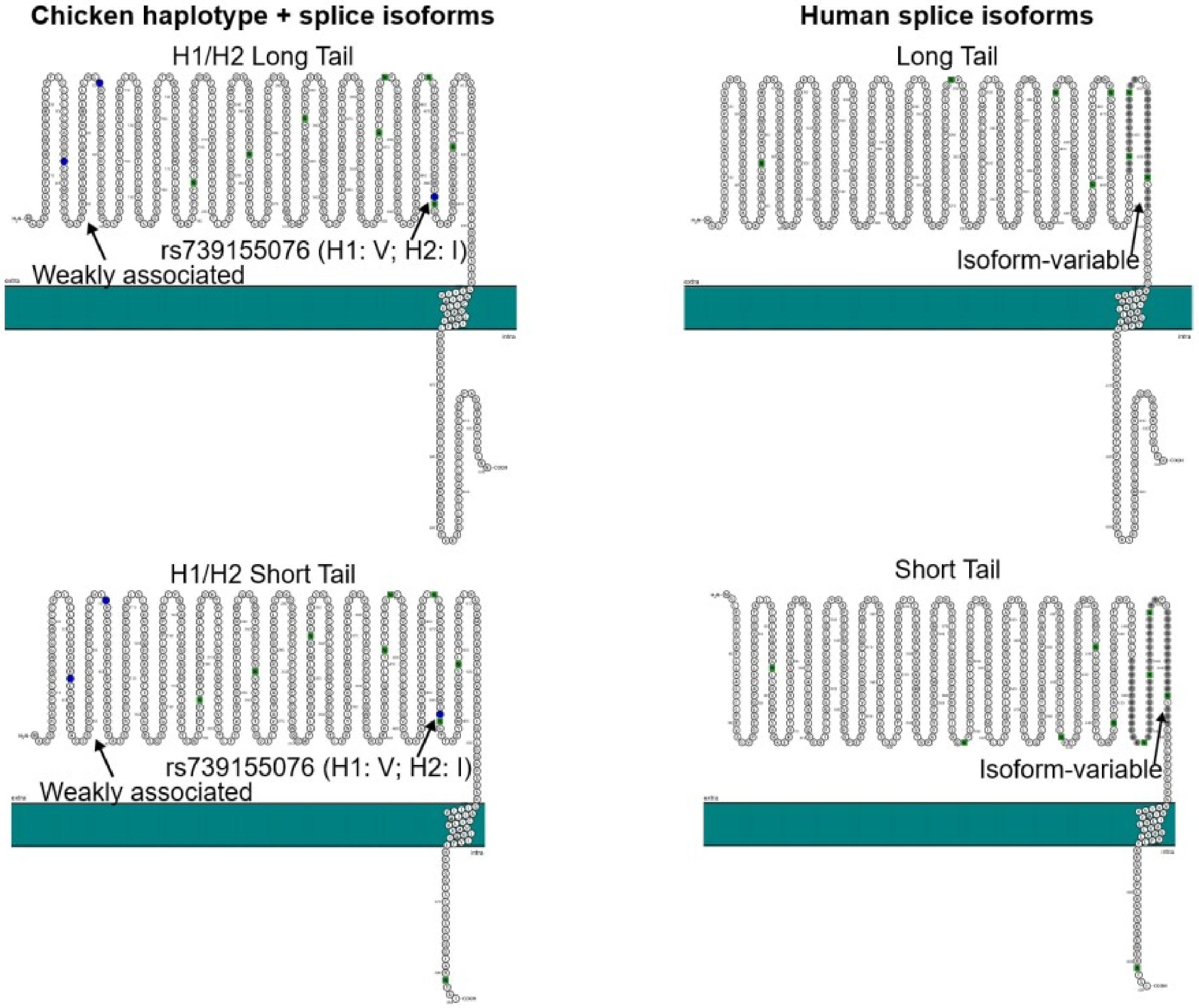

Supplementary Figure S3: Membrane topology of chicken MCAM (CD146) haplotypes and splice isoforms compared with human isoforms. Predicted chicken haplotype classes H1 and H2, each observed with alternative long (L) and short (S) cytoplasmic tail architectures (left panel). Human CD146 are shown for comparison (right panel). The chicken H1/H2 haplotype class assignment is defined on the coding variant rs739155076 (V482I), and the haplotype-associated amino acids are indicated. Regions annotated as isoform-variable in human reflect differences in membrane-proximal extracellular segments among retrieved sequences. Topology schematics were generated with Protter from chicken (long, NP_001004768.1; short, BAA07563.1) and human isoforms (long, XP_054224795.1; short, XP_054224797.1).

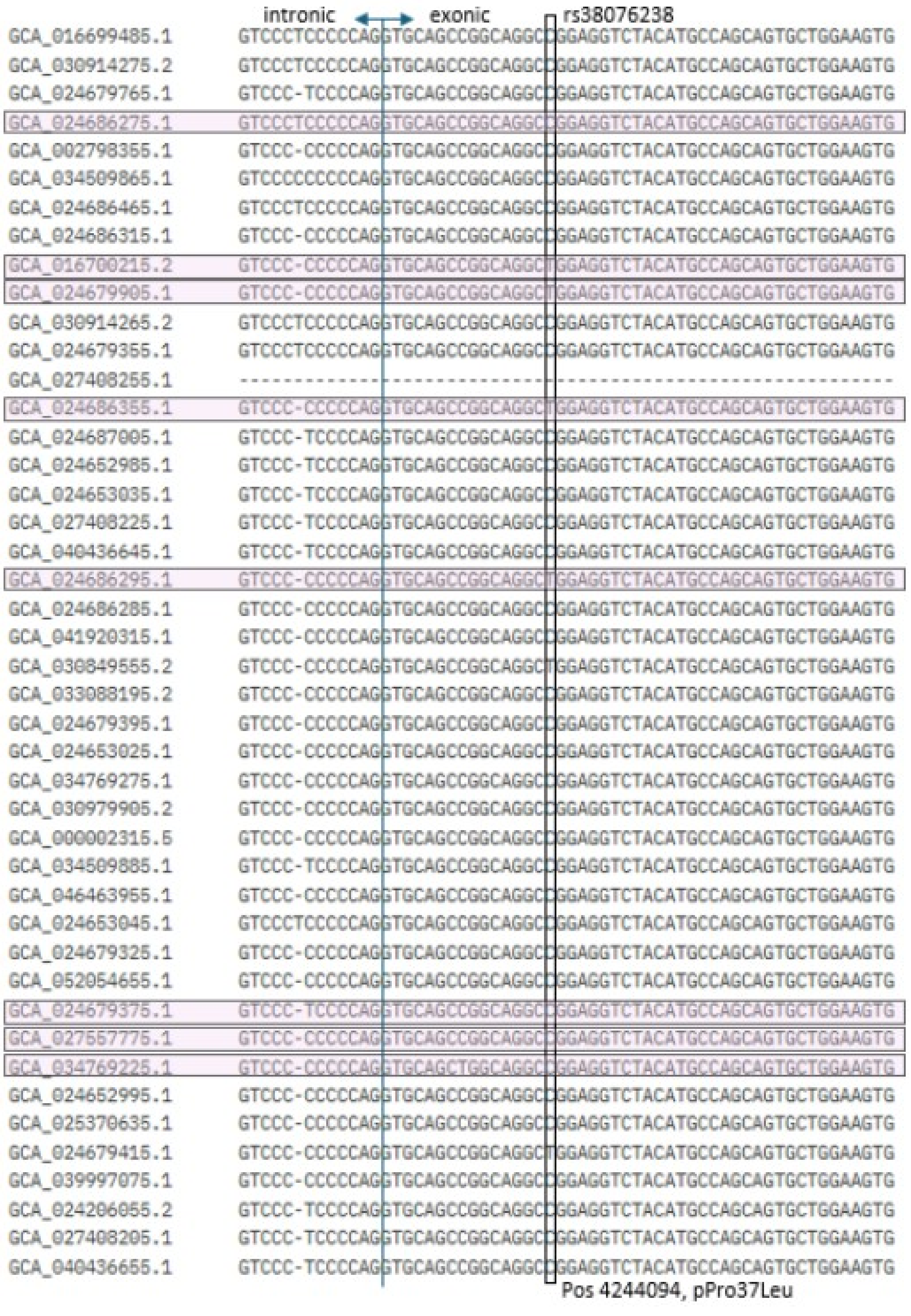

Supplementary Figure S4: Exon 2 alignment of chicken MCAM (CD146) highlighting the P37L variant (rs38076238). Alignment of sequences spanning positions 424,068–424,125 (exon 2) across 45 chicken genomes. Exonic and intronic regions are indicated, and the SNP corresponding to the P37L substitution is marked. Haplotype assignments (H1, white; H2, purple) are shown for reference. The P37L variant does not segregate consistently with H1 and H2 haplotypes.

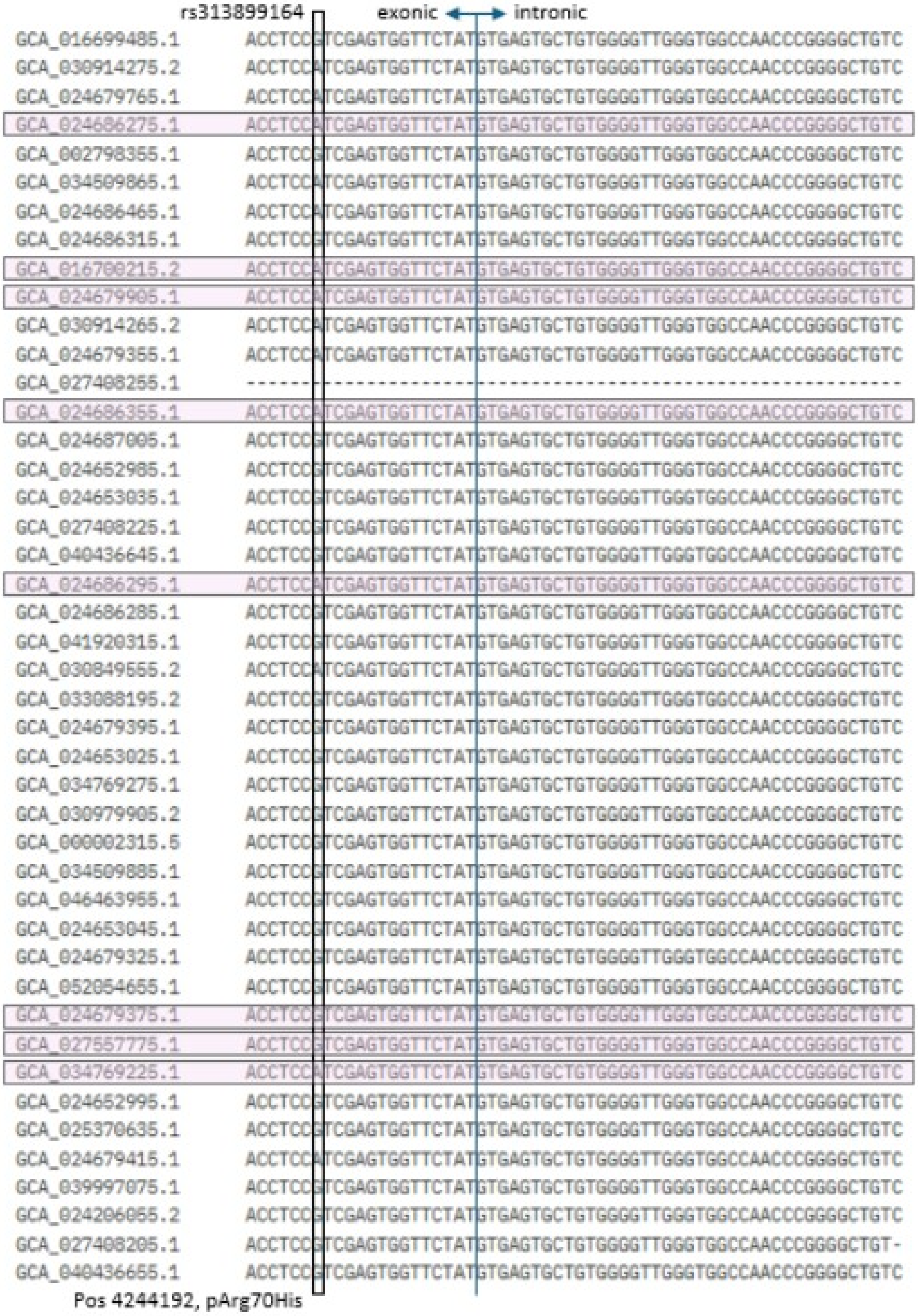

Supplementary Figure S5: Exon 3 alignment of chicken MCAM (CD146) highlighting the P70H variant (rs313899164). Alignment of sequences spanning positions 424,186–424,245 (exon 3) across 45 chicken genomes. Exonic and intronic regions are indicated, and the SNP corresponding to the P70H substitution is marked. Haplotype assignments (H1, white; H2, purple) are shown for reference. The P70H variant does not segregate consistently with H1 and H2 haplotypes.

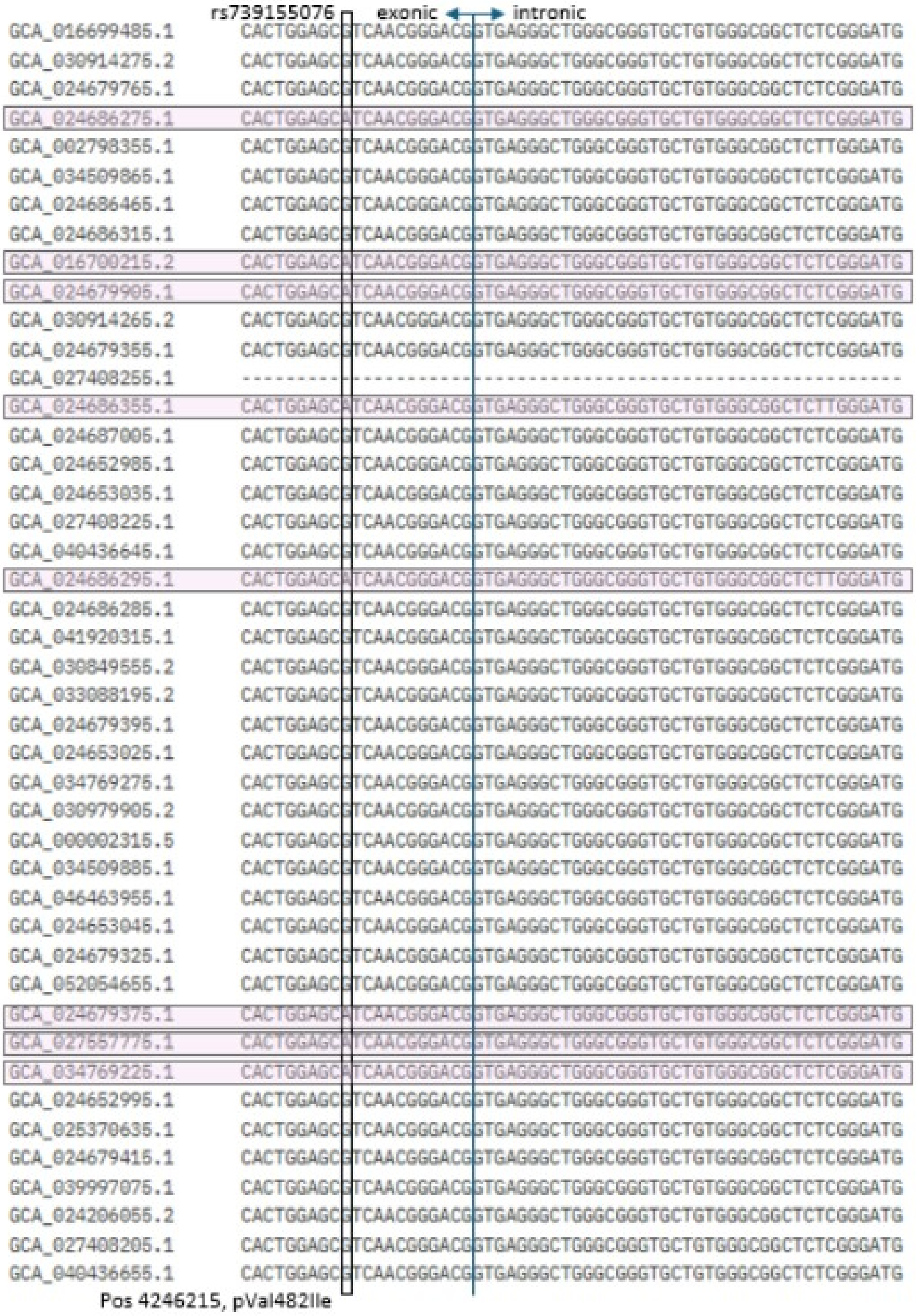

Supplementary Figure S6: Chicken MCAM (CD146) alignment highlighting the V482I variant (rs739155076). Alignment of sequences spanning positions 424,206–424,265 (exon 13) across 45 chicken genomes. Exonic and intronic regions are indicated, and the SNP position corresponding to the V482I substitution is marked. Sequences carrying the H1 haplotype are shown in white, and those carrying the H2 haplotype are shown in purple.
